# Auditory, Visual and Audiovisual Speech Processing Streams in Superior Temporal Sulcus

**DOI:** 10.3389/fnhum.2017.00174

**Published:** 2017-04-07

**Authors:** Jonathan H. Venezia, Kenneth I. Vaden, Feng Rong, Dale Maddox, Kourosh Saberi, Gregory Hickok

**Affiliations:** ^1^VA Loma Linda Healthcare SystemLoma Linda, CA, USA; ^2^Department of Otolaryngology—Head and Neck Surgery, Medical University of South CarolinaCharleston, SC, USA; ^3^Department of Cognitive Sciences, Center for Cognitive Neuroscience and Engineering, University of CaliforniaIrvine, CA, USA

**Keywords:** audiovisual speech, superior temporal sulcus, fMRI, visual motion, functional gradient

## Abstract

The human superior temporal sulcus (STS) is responsive to visual and auditory information, including sounds and facial cues during speech recognition. We investigated the functional organization of STS with respect to modality-specific and multimodal speech representations. Twenty younger adult participants were instructed to perform an oddball detection task and were presented with auditory, visual, and audiovisual speech stimuli, as well as auditory and visual nonspeech control stimuli in a block fMRI design. Consistent with a hypothesized anterior-posterior processing gradient in STS, auditory, visual and audiovisual stimuli produced the largest BOLD effects in anterior, posterior and middle STS (mSTS), respectively, based on whole-brain, linear mixed effects and principal component analyses. Notably, the mSTS exhibited preferential responses to multisensory stimulation, as well as speech compared to nonspeech. Within the mid-posterior and mSTS regions, response preferences changed gradually from visual, to multisensory, to auditory moving posterior to anterior. *Post hoc* analysis of visual regions in the posterior STS revealed that a single subregion bordering the mSTS was insensitive to differences in low-level motion kinematics yet distinguished between visual speech and nonspeech based on multi-voxel activation patterns. These results suggest that auditory and visual speech representations are elaborated gradually within anterior and posterior processing streams, respectively, and may be integrated within the mSTS, which is sensitive to more abstract speech information within and across presentation modalities. The spatial organization of STS is consistent with processing streams that are hypothesized to synthesize perceptual speech representations from sensory signals that provide convergent information from visual and auditory modalities.

## Introduction

The superior temporal sulcus (STS) is activated during a variety of perceptual tasks including audiovisual integration (Beauchamp et al., 2004b; Amedi et al., 2005), speech perception (Binder et al., 2000, 2008; Hickok and Poeppel, 2004, 2007; Price, 2010), and biological motion perception (Allison et al., 2000; Grossman et al., 2000, 2005; Grossman and Blake, 2002; Beauchamp et al., 2003; Puce and Perrett, 2003). It has been widely established that auditory speech perception is influenced by visual speech information (Sumby and Pollack, 1954; McGurk and MacDonald, 1976; Dodd, 1977; Reisberg et al., 1987; Callan et al., 2003), which is represented in part within biological motion circuits that specify the shape and position of vocal tract articulators. This high-level visual information is hypothesized to interact with auditory speech representations in the STS (Callan et al., 2003). Indeed, the STS is well-positioned to integrate auditory and visual inputs as it lies between visual association cortex in the posterior lateral temporal region (Beauchamp et al., 2002) and auditory association cortex in the superior temporal gyrus (Rauschecker et al., 1995; Kaas and Hackett, 2000; Wessinger et al., 2001). In nonhuman primates, polysensory fields in STS have been shown to receive convergent input from unimodal auditory and visual cortical regions (Seltzer and Pandya, 1978, 1994; Lewis and Van Essen, 2000) and these fields contain auditory, visual and bimodal neurons (Benevento et al., 1977; Bruce et al., 1981; Dahl et al., 2009). Furthermore, human functional neuroimaging evidence supports the notion that the STS is a multisensory convergence zone for speech (Calvert et al., 2000; Wright et al., 2003; Beauchamp et al., 2004a, 2010; Szycik et al., 2008; Stevenson and James, 2009; Stevenson et al., 2010, 2011; Nath and Beauchamp, 2011, 2012).

**Figure 1:**
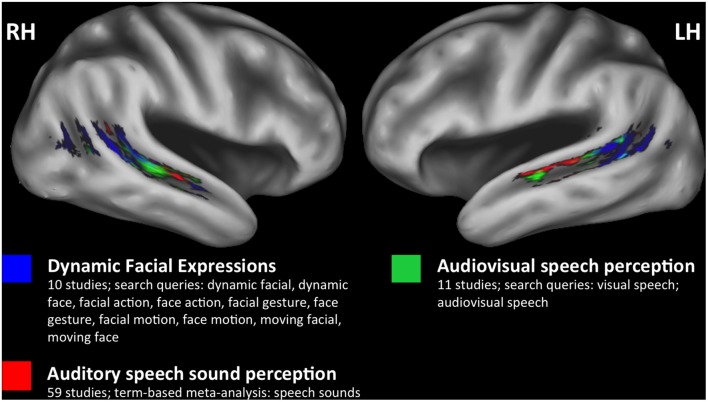
**Meta-analyses.** A posterior to anterior gradient of effects related to visual and auditory speech information in bilateral superior temporal sulcus (STS). Three separate meta-analyses were performed using NeuroSynth (http://neurosynth.org) to identify studies that only included healthy participants and reported effects in STS. Two custom meta-analyses (dynamic facial expressions, audiovisual speech) and one term-based meta-analysis (speech sounds) were performed (see color key for details). The FDR-corrected (*p* < 0.01) reverse inference Z-statistic maps for each meta-analysis were downloaded from NeuroSynth for plotting. Results from dynamic facial expressions (blue), audiovisual speech (green), and speech sounds (red) meta-analyses are plotted on the study-specific template in MNI space (see “Study-Specific Anatomical Template” Section) and restricted to an STS region of interest to highlight the spatial distribution of effects within the STS (see “STS Region of Interest Analysis” Section).

However, it remains unclear what role, if any, biological-motion-sensitive regions of the STS play in multimodal speech perception. By and large, facial motion—including natural facial motion (Puce et al., 1998), movements of facial line drawings (Puce et al., 2003), and point-light facial motion (Bernstein et al., 2011)—yield activation quite posteriorly in the STS, a location that is potentially distinct from auditory and visual speech-related activations. The results of a meta-analysis (Figure 1) performed using NeuroSynth (Yarkoni et al., 2011) show peak activations for dynamic facial expressions, audiovisual speech, and auditory speech sounds that are distributed posterior-to-anterior along the STS, respectively. Previous work has established a similar visual-to-auditory gradient within regions of the STS that respond to audiovisual speech (Wright et al., 2003), and the gradient in Figure 1 further suggests that neural populations near the posterior STS are active in visual processing related to facial and biological motion perception. It is hypothesized that posterior-visual STS regions facilitate the extraction of abstract properties from biological motion stimuli (e.g., action class or action goal), defined by their invariance to specific features including motion kinematics, image size, or viewpoint (Lestou et al., 2008; Grossman et al., 2010). Likewise, facial motion computations in posterior STS could contribute to abstracted speech representations (Bernstein and Liebenthal, 2014), although the relationship between biological motion and audiovisual speech systems in the STS has not been completely characterized.

A challenge to characterizing spatially proximal and functionally related multimodal speech systems within the STS is that neuroimaging studies of visual speech processing typically factor out activation to nonspeech facial-motion control stimuli (Campbell et al., 2001; Okada and Hickok, 2009; Bernstein et al., 2011). This strategy could limit sensitivity to cortical regions that respond to both speech and nonspeech facial motion, outlined above as supporting action encoding and other pre-linguistic perceptual processes. Similar arguments have been made regarding the interpretation of contrast-based neuroimaging studies in the auditory speech domain (Okada et al., 2010; Stoppelman et al., 2013). Moreover, visual speech/lipreading studies have not directly examined visual-speech-specific responses with respect to functionally defined auditory and/or multimodal speech networks. The goal of the present fMRI experiment was to more completely characterize modality-dependent and independent responses to speech, particularly within the STS. As such, we set out to map the network of auditory, visual and audiovisual^1^ speech processing regions of the STS in unprecedented detail. Our investigation was guided by the following questions: (1) Does the anterior-posterior gradient of auditory and visual responses in STS observed across studies (Figure 1) replicate within a single, independent group of participants? If so (2) at what level of processing do speech-specific representations emerge in the STS; in particular, do posterior-visual regions of the STS play a role in speech processing? Participants were presented with auditory and visual speech (consonant-vowel (CV) syllables) and nonspeech (spectrally rotated syllables, nonspeech facial gestures) to enable measurement of modality-dependent and independent responses in the STS that are hypothesized to contribute to speech recognition.

## Materials and Methods

### Participants

This study was approved by the UC Irvine Institutional Review Board and carried out according to the Declaration of Helsinki. All participants gave written informed consent prior to their participation. Twenty (three females) right-handed native English speakers between 18 and 30 years of age participated in the study. All volunteers had normal or corrected-to-normal vision, normal hearing by self-report, no known history of neurological disease, and no other contraindications for MRI. Two participants were excluded from MRI analysis leaving *N* = 18 for the imaging analysis (see below).

### Stimuli and Procedure

#### Stimuli

**Figure 2:**
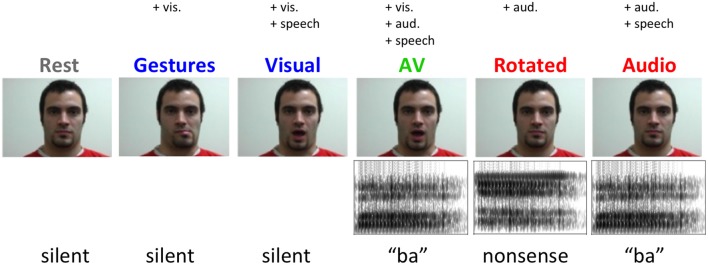
**Example stimuli from each experimental condition**.

Six two-second video clips were recorded for each of five experimental conditions featuring a single male actor shown from the neck up (Figure 2). In three speech conditions—auditory speech (A), visual speech (V), and audiovisual speech (AV)—the stimuli were six visually distinguishable CV syllables (\ba\, \tha\, \va\, \bi\, \thi\, \vi\). In the A condition, clips consisted of a still frame of the actor’s face paired with auditory recordings of the syllables (44.1 kHz, 16-bit resolution). In the V condition, videos of the actor producing the syllables were presented without sound (30 frames/s). In the AV condition, videos of the actor producing the syllables were presented simultaneously with congruent auditory recordings. There were also two non-speech conditions—spectrally rotated speech (R) and nonspeech facial gestures (G). In the R condition, spectrally inverted (Blesser, 1972) versions of the auditory syllable recordings were presented along with a still frame of the actor. Rotated speech stimuli were created from the original auditory syllable recordings by first bandpass filtering (100–3900 Hz) and then spectrally inverting about the filter’s center frequency (2000 Hz). Spectral rotation preserves the spectrotemporal complexity of speech, producing a stimulus that is acoustically similar to clear speech but unintelligible (Scott et al., 2000; Narain et al., 2003; Okada et al., 2010) or, in the case of sublexical speech tokens, significantly less discriminable (Liebenthal et al., 2005). In the G condition, the actor produced the following series of nonspeech lower-face gestures without sound: partial opening of the mouth with leftward deviation, partial opening of mouth with rightward deviation, opening of mouth with lip protrusion, tongue protrusion, lower lip biting and lip retraction. These gestures contain movements of a similar extent and duration as those used to produce the syllables in the speech conditions, but cannot be construed as speech (Campbell et al., 2001). A rest condition was included consisting of a still frame of the actor with no sound. All auditory speech stimuli were bandpass filtered to match the bandwidth of the rotated speech stimuli. All auditory stimuli were normalized to equal root-mean-square amplitude.

Twelve-second blocks were created by concatenating the individual video clips in each experimental condition. Each block contained all six of the clips from that condition (i.e., all six CV syllables, all six rotated CV syllables, or all six nonspeech facial gestures). The clips were concatenated in random order to form 35 distinct blocks in each condition. Five additional “oddball” blocks were created for each condition including rest, consisting of five within-condition clips and a single oddball clip from one of the other conditions (e.g., an oddball block might contain five A clips and a single AV clip). Oddball clips were placed at random in one of the five positions following the first clip in the block. An oddball could deviate from the standards either visually (e.g., a V clip in a G block), acoustically (e.g., an A clip in an R block), or both (e.g., an AV clip in an R block or an A clip in a V block). Each of these types of deviation occurred with equal frequency so that participants would attend equally to auditory and visual components of the stimuli. We selected the oddball task because it did not force participants to explicitly categorize or identify individual stimuli—particularly speech sounds—within a block. The oddball task asked participants to detect deviance on the basis of stimulus condition rather than stimulus identity *per se*. This low-level task ensured that speech-related activations were not contaminated by linguistic and/or verbal working memory demands unrelated to sensory-perceptual processing.

##### Motion energy in visual speech vs. nonspeech facial gestures

Bernstein et al. (2011) point out that speech and nonspeech facial gestures such as those used in the present study may not be well-matched on a number of low-level characteristics including total motion energy. To test this, we computed an estimate of the total motion energy in our V and G stimuli as follows. For each video clip, a frame-by-frame estimate of the vertical and horizontal optical flow velocity was calculated using the Horn-Schunck algorithm (Horn and Schunck, 1981) implemented in MATLAB. The total motion energy in each clip was computed as the root-mean-square optical flow velocity across both image dimensions and all frames. A condition-level estimate of the total motion energy was computed by summing the estimated motion energy across all six clips in a given condition. Using this approach, we found that nonspeech facial gestures (G) had 33% more total motion energy than speech gestures (V). As such, we should expect that brain regions sensitive to motion energy would activate more to G than V.

#### Procedure

Functional imaging runs consisted of pseudo-random presentation of 21 blocks, three from each condition along with three rest blocks and three oddball blocks. Blocks were separated by a 500 ms inter-block interval during which a black fixation cross was presented against a gray background. Participants were instructed to press a button each time an oddball was detected. The experiment started with a short practice session inside the scanner during which participants were exposed to a single block from each condition including a rest block and an oddball block. Participants were then scanned for ten functional runs immediately followed by acquisition of a high-resolution T1 anatomical volume. Auditory stimuli were presented through an MR compatible headset (ResTech) and stimulus delivery and timing were controlled using Cogent software^2^ implemented in Matlab 6 (Mathworks Inc., Natick, MA, USA).

### Image Acquisition

MR images were obtained on a Philips Achieva 3T (Philips Medical Systems, Andover, MA, USA) fitted with an 8-channel SENSE receiver/head coil, at the Research Imaging Center facility at the University of California, Irvine. We collected a total of 1090 echo planar imaging (EPI) volumes over 10 runs using single pulse Gradient Echo EPI (matrix = 112 × 110, TR = 2.5 s, TE = 25 ms, size = 1.957 × 1.957 × 1.5 mm, flip angle = 90). Forty-Four axial slices provided whole brain coverage. Slices were acquired sequentially with a 0.5 mm gap. After the functional scans, a high-resolution anatomical image was acquired with a magnetization prepared rapid acquisition gradient echo (MPrage) pulse sequence in the axial plane (matrix = 240 × 240, TR = 11 ms, TE = 3.54 ms, size = 1 × 1 × 1 mm).

### Data Analysis

#### Behavioral Data Analysis

The Signal Detection Theory measure *d′* was calculated to determine performance on the oddball detection task (Green and Swets, 1966). A hit was defined as a positive response (button press) to an oddball block, while a false alarm was defined as a positive response to a non-oddball block. The hit rate (H) was calculated as the number of hits divided by the total number of oddball blocks, while the false alarm rate (F) was calculated as the number of false alarms divided by the number of non-oddball blocks, and *d′* was calculated as:

(1) $$d^{\prime }\;=\;\Phi^{-\text{1}}\left(H\right)-\Phi^{-\text{1}}\left(F\right)$$

where Φ is the standard normal cumulative distribution function. Participants with a *d′* greater than 1.5 standard deviation below the group mean were excluded from further analysis (*N* = 2; see “Results” Section). We also calculated H separately for each oddball type (A, V, AV, R, G). These condition-specific hit rates were entered in a repeated measures ANOVA with Greenhouse/Geisser correction.

#### Neuroimaging Analysis

Minus the two participants excluded for poor behavioral performance, the total sample size for neuroimaging analysis was *N* = 18.

##### Study-specific anatomical template

A study-specific anatomical template image was created using symmetric diffeomorphic registration (SyN) in the Advanced Normalization Tools (ANTS v2.0.0/2.1.0) software (Avants and Gee, 2004; Avants et al., 2008). Each participant’s T1 anatomical image was submitted to the template-construction processing stream in ANTS (buildtemplateparallel.sh), which comprises rigid and SyN registration steps. For SyN, we used a cross correlation similarity metric (Avants et al., 2011) with a three-level multi-resolution registration with 50 × 70 × 10 iterations. The whole-head template was skull stripped in ANTS (antsBrainExtraction.sh) and a brain+cerebellum mask of the skull-stripped template was inverse warped to each participant’s native space and used to skull strip the individual participant T1 images. These skull-stripped images were then re-registered to the skull-stripped template using SyN. The resulting study-specific anatomical template was then aligned to the MNI-space ICBM 152 nonlinear atlas version 2009c (Fonov et al., 2009, 2011) using a 12-parameter affine registration in ANTS. The ICBM atlas, which had better tissue contrast than our study-specific template image, was diffeomorphically warped (SyN) to the study-specific template in MNI space. The warped version of the ICBM atlas was used to plot the functional data.

##### Preprocessing

Preprocessing of the functional data was performed using AFNI (v16.0.11) software^3^. For each run, slice timing correction was performed followed by realignment (motion correction) and coregistration of the EPI images to the high resolution anatomical image. Spatial normalization was then performed by applying the set of rigid, diffeomorphic, and affine transformations mapping each participant’s anatomical image to the study-specific template in MNI space (antsApplyTransforms, linear interpolation). Images were then spatially smoothed with an isotropic 6-mm full-width half-maximum (FWHM) Gaussian kernel, and each run was scaled to have a mean of 100 across time at each voxel.

##### First level analysis

First level regression analysis (AFNI 3dDeconvolve) was performed in individual subjects. To create the regressors of interest, a stimulus-timing vector was created for each experimental condition and convolved with a model hemodynamic response function. The “still face” rest condition was not modeled explicitly and was thus included in the baseline term. An additional 12 regressors corresponding to motion parameters determined during the realignment stage of preprocessing along with their temporal derivatives were entered into the model. Oddball blocks were modeled as a single regressor of no interest. Individual time points were censored from analysis when more than 10% of in-brain voxels were identified as outliers (AFNI 3dToutcount) or when the Euclidean norm of the motion derivatives exceeded 0.4.

##### Group analysis

A second-level analysis of variance was performed on the first-level parameter estimates (henceforth “percent signal change” (PSC)) from each participant, treating “participant” as a random effect. Activation images (mean PSC) and statistical parametric maps (*t*-statistics) were created for each individual condition. Significantly active voxels were defined as those for which *t*-statistics exceeded the *p* < 0.005 level with a cluster extent threshold of 185 voxels. This cluster threshold held the family-wise error rate (FWER) less than 0.05 as determined by Monte Carlo simulation using AFNI 3dClustSim with padding to minimize edge effects (Eklund et al., 2016). Estimates of smoothness in the data were drawn from the residual error time series for each participant after first-level analysis (AFNI 3dFWHMx). These estimates were averaged across participants separately in each voxel dimension for input to 3dClustSim.

To identify multisensory regions at the group level, we performed the conjunction A∩V, and to identify regions sensitive to facial motion at the group level, we performed the conjunction V∩G. Conjunctions were performed by constructing minimum *t*-maps (e.g., minimum T score from [A, V] at each voxel) and these maps were thresholded at *p* < 0.005 with a cluster extent threshold of 185 voxels (FWER < 0.05) as for individual condition maps. This tests the “conjunction null” hypothesis (Nichols et al., 2005). We also performed contrasts for activations greater for speech than nonspeech, matched for input modality: A > R and V > G.

##### STS region of interest analysis

An STS ROI was generated from the TT_desai_ddpmaps probabilistic atlas distributed with AFNI. The atlas, which is based on Freesurfer demarcation of the STS in 61 brains (see, Liebenthal et al., 2014), shows for each voxel the percentage of brains in which that voxel was included in the STS. Left and right hemisphere probabilistic maps of the STS were thresholded at 30% to create a binary mask for ROI analysis. The ROI mask, which was originally aligned to the TT_N27 (Colin) template brain distributed with AFNI, was then aligned to our study-specific template in MNI space by first warping the TT_N27 template to our study-specific template using a 12-parameter affine transformation in ANTS, and then applying the transformation matrix to the STS binary mask image using nearest neighbor interpolation. The left and right STS ROIs were then subdivided splitting the STS into eight equal-length subregions along the anterior-posterior axis. The centers of mass of each STS subregion are provided in Table 1.

**Table 1:** **Centers of mass of superior temporal sulcus (STS) subregions (MNI coordinates)**.

	Left STS			Right STS		
**Subregion number**	*x*	*y*	*z*	*x*	*y*	*z*
(Anterior) 1	−52	−4	−17	52	−4	−17
2	−54	−13	−11	53	−15	−10
3	−54	−22	−7	52	−24	−5
4	−54	−32	−2	52	−32	0
5	−54	−42	4	51	−41	10
6	−50	−52	15	50	−50	18
7	−46	−62	21	47	−58	18
(Posterior) 8	−43	−71	23	45	−65	22

###### Generalized linear mixed model: auditory, visual, speech effects on BOLD

A generalized linear mixed model (GLMM) regression analysis was performed to characterize the extent to which BOLD contrast changed within STS subregions during auditory, visual and speech presentations. For each participant, block-by-block PSC estimates for each condition were extracted from each of the eight STS subregions in each hemisphere using “Least Squares—Separate” (LS-S) regression (Mumford et al., 2012). In the LS-S regression (AFNI 3dLSS), the model included one regressor of interest modeling a single block from a given condition (e.g., V), and five nuisance regressors modeling: (1) all other blocks in the condition of interest (e.g., V); and (2) all blocks in the remaining conditions (e.g., A, AV, R, G). Run-level baseline and drift terms were included in order to remove global signal differences and differential trends across runs. Repeating this for each block in each condition produced an LS-S “time series” with block-level BOLD estimates that served as input to the GLMM. The GLMM parameterized the five conditions (A, V, AV, R, G) to capture modality-dependent BOLD changes. First, an auditory parameter (AP) was coded as AP = 1 for the A, AV, and R conditions, and AP = 0 for the V and G conditions. Second, a visual parameter (VP) was coded as VP = 1 for the V, AV, and G conditions, and VP = 0 for the A and R conditions. Finally, a speech parameter (SP) was coded as SP = 1 for the A, V and AV conditions, and SP = 0 for the R and G conditions. The GLMM including fixed effects for AP, VP and SP, as well as participant-level random intercept and random slope terms for AP, VP and SP, was fit to the block-level BOLD data separately for each STS subregion in each hemisphere. The predictor variables were scaled within participant and region (*m* = 0, sd = 1) to account potentially unbalanced observations. Separately for each participant, observations with extreme BOLD values were excluded from the model based on the following formula for outlier detection:

(2) $$C=\alpha *\sqrt{\pi /2}*MAD;\alpha =\Phi^{-1}\left(1-0.001/N\right)$$

where C is the outlier cut-off, MAD is the median absolute deviation, Φ is the standard normal cumulative distribution function, and *N* is the number of time points (blocks). The GLMM equation can be expressed as follows:

(3) $$BOLD_{s,c}\;\sim \;AP_{s,c}+VP_{s,c}+SP_{s,c}\;+\left(\text{1}+AP_{s,c}+VP_{s,c}+SP_{s,c}|s\right)+error,$$

where *s* stands for subject and *c* for condition.

Non-parametric significance tests were performed after calculating *t*-scores for AP, VP and SP effects. Because the ROI statistic results were not spatially independent, family-wise error corrected significance was calculated by permuting predictor variables without replacement. Empirical null distributions of *t*-scores were computed by randomly permuting condition order and recalculating *t*-scores (10,000 reshuffled samples). Each permutation was applied to AP, VP and SP conditions identically to preserve covariance among the parameters. Furthermore, the same permutation was used for each STS subregion to preserve spatial dependencies. The dependent variable (BOLD) was not reordered. After each permutation, the maximum and minimum *t*-scores across STS subregions were used to create empirical *max-t* and *min-t* distributions for each fixed effect (AP, VP, SP). Observed *t*-scores (*t*_obs_) were compared to the *max-t* or *min-t* distributions to calculate one tailed *p*-values: *P(max-t > t_obs_)* for positive *t*-scores, or *P(min-t < t_obs_)* for negative scores. Tests with *p* < 0.05 were considered significant with FWER < 0.05 across all STS subregions (Nichols and Holmes, 2002).

###### Principal component analysis

To summarize changes in the pattern of responses across conditions throughout the STS, we conducted a principal component analysis on the group activation images for each of the five experimental conditions. In the PCA, each voxel of the left or right STS was considered as a separate variable, and each experimental condition was considered as a separate observation. The first two principal components were extracted. Each component yielded a score for each experimental condition, where the pattern of scores demonstrated how patterns of activation across conditions separated along that principal dimension. Each component also yielded a set of coefficients across voxels, where the sign of the coefficient determined which conditions that voxel preferred and the magnitude of the coefficient determined the extent to which that voxel’s activation followed the pattern described by the condition scores.

###### *Post hoc* analysis of visual STS subregions

The results of the GLMM analysis (see “GLMM Region of Interest Results” Section) revealed several posterior STS subregions in each hemisphere that showed a significant preference for stimuli containing visual information (V, AV, G) vs. only auditory information (A, R). For the five visually responsive/preferring subregions (three right hemisphere, two left hemisphere), we performed a repeated-measures ANOVA comparing PSC in V, AV and G (Greenhouse-Geisser correction for violations of sphericity; *α* = 0.05, uncorrected). In STS regions that did not exhibit significant univariate differences for V, AV and G, multivariate pattern classification analysis (MVPA) was performed to determine whether these conditions could be distinguished in terms of spatial patterns of activity. This analysis was crucial because regions that activated significantly during visual presentations, yet did not distinguish between visual conditions on the basis of univariate activation, could have nonetheless carried important, condition-specific information in multivariate activation patterns (Mur et al., 2009). MVPA was achieved using a support vector machine (SVM; MATLAB Bioinformatics Toolbox v3.1, The MathWorks, Inc., Natick, MA, USA) as the pattern classification method. Two pairwise classifications were performed. First, activity patterns were used to discriminate between two different types of facial motion (V vs. G). Second, activity patterns were used to differentiate between visual and audiovisual trials with identical visual information (V vs. AV). Both MVPA tests were conducted on BOLD time series data in native space. The STS subregions in the group anatomical space were spatially transformed into native space using the inverse of the transformations mapping each participant’s anatomical image to the study-specific template (ANTS, nearest neighbor interpolation).

Inputs to the classifier were estimates of activation to each block calculated using LS-S regression as described above. LS-S coefficients representing all 15 blocks for each condition were calculated and stored with appropriate run labels at each voxel. Prior to classification, LS-S coefficients for each ROI were *z*-scored across voxels for each block, effectively removing mean amplitude differences across blocks (Mumford et al., 2012; Coutanche, 2013).

We performed SVM classification on the LS-S data using a leave-one-out cross validation approach within-subject (Vapnik, 1999). In each iteration, we used data from 9 of the 10 functional scan runs to train an SVM classifier and then used the trained classifier to test the data from the remaining run. The SVM-estimated condition labels for the testing data set were then compared with the real labels to compute classification sensitivity. For each pairwise classification, one condition was arbitrarily defined as signal and the other as noise. A classifier hit was counted when the SVM-estimated condition label matched the real condition label for the “signal” condition, and a false alarm was counted when the SVM-estimated label did not match the real condition label in the “noise” condition. A measure of sensitivity, *d′*, was calculated following the formula for a yes-no experiment (equation 1, above). Classification *d′* for each subject was derived by averaging the *d′* scores across all leave-one-out runs, and an overall *d′* was computed by averaging across subjects for each pairwise classification.

Classification *d′* scores were evaluated statistically using a nonparametric bootstrap method (Lunneborg, 2000). Classification procedures were repeated 10,000 times for each pairwise classification within each individual data set, with the condition labels reshuffled per repetition. This provided an empirical null distribution of *d′* for each subject and pairwise classification. A bootstrap-T approach was used to assess the significance of the classification *d′* across participants. For each repetition of the bootstrap, a *t*-test of the bootstrapped *d′* scores across all subjects against the ideal chance *d′* score (zero) was performed. The observed *t*-score (*t*_obs_) obtained from the true data was then statistically tested against the empirical null distribution of *t*-scores (*t*_null_). A *p*-value was calculated as *P*(*t*_null_ > *t*_obs_), where *p* < 0.05 determined that *d′* was significantly greater than chance across subjects.

## Results

### Behavior

Based on their performance in the condition-oddball detection task, two participants were below the behavioral cut off and were excluded from further analysis (*d′* = 1.85, hits = 14/30, false alarms = 4/150; and *d′* = 2.13, hits = 14/30, false alarms = 2/150). The remaining eighteen participants performed well on the task (mean *d′* = 3.40 ± 0.14 SEM, mean hits = 26/30 ± 0.56 SEM, mean false alarms = 3/150 ± 0.84 SEM), which indicated that they attended to both auditory and visual components of the stimuli. Among the participants whose performance exceeded the behavioral cutoff, there was not a significant difference in hit rate across conditions (*F*_(2.3,38.7)_ = 2.07, *p* = 0.13).

### Neuroimaging

#### Whole-Brain Results

**Figure 3:**
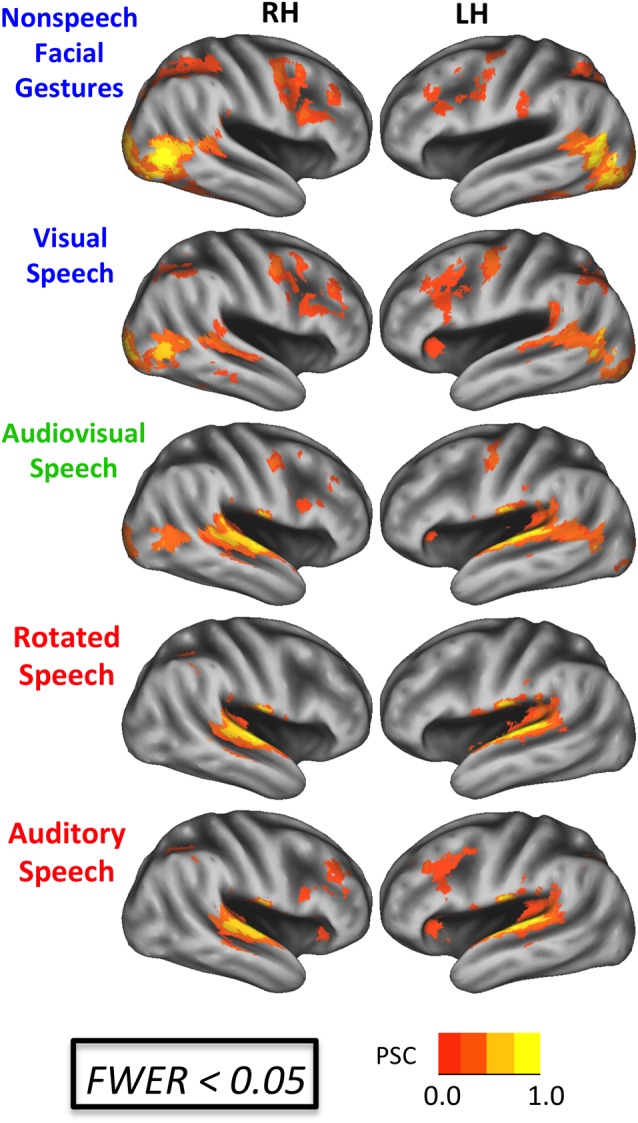
**BOLD effects during each experimental condition.** Results are shown on an inflated surface rendering of the study-specific template in MNI space. Top: speech conditions (A, V, AV). Bottom: nonspeech conditions (R, G). All maps thresholded at an uncorrected voxel-wise *p* < 0.005 with a cluster threshold of 185 voxels (family-wise error rate (FWER) corrected *p* < 0.05). PSC, percent signal change.

**Figure 4:**
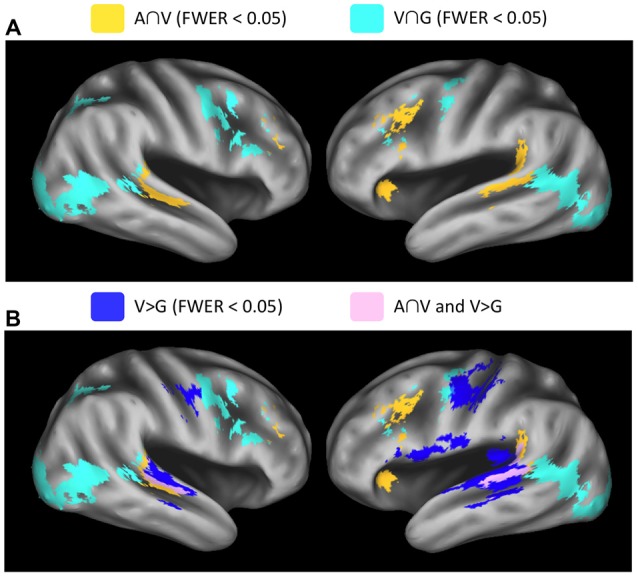
**Whole-brain conjunction analyses. (A)** Group-level conjunction results plotted on an inflated surface rendering of the study-specific template in MNI space. Significant responses during visual speech and nonspeech facial gestures (V∩G, teal) were observed in the posterior STS. Significant responses during auditory speech and visual speech (A∩V, yellow) were observed the middle STS (mSTS). **(B)** Conjunction analyses are plotted together with the contrast V vs. G (blue), which highlights regions that activate preferentially to visual speech vs. nonspeech facial gestures. These visual-speech-specific regions fall anterior to V∩G in the STS and overlap strongly with A∩V (pink). All maps were thresholded at voxel-wise *p* < 0.005 (uncorrected) with a cluster-extent threshold of 185 voxels (FWER corrected *p* < 0.05).

Activation maps for each of the five experimental conditions relative to rest are shown in Figure 3 (FWER < 0.05). Visual facial gestures (V, AV, G) activated bilateral primary and secondary visual cortices, lateral occipital-temporal visual regions, inferior and middle temporal gyri, and posterior STS. Conditions containing auditory information (A, AV, R) activated supratemporal auditory regions, the lateral superior temporal gyrus, and portions of the STS bilaterally. All conditions except for R activated bilateral inferior frontal regions. We tested directly for voxels showing an enhanced response to intelligible speech by computing the contrasts A > R and V > G. The A > R contrast (not displayed) did not yield any significant differences at the group level. Although this is not consistent with previous imaging work (Scott et al., 2000; Narain et al., 2003; Liebenthal et al., 2005; Okada et al., 2010), we believe that our use of sublexical stimuli may have contributed to this null result. The V > G contrast yielded a visual speech network consistent with previous work (Campbell et al., 2001; Callan et al., 2004; Okada and Hickok, 2009; Bernstein et al., 2011; Hertrich et al., 2011), including bilateral STS, left inferior frontal gyrus, and a host of inferior parietal and frontal sensory-motor brain regions (Figure 4B).

Results from the conjunction analyses demonstrated overlapping Auditory and Visual speech effects (A∩V) in STS/STG locations that were anterior with respect to Visual speech and nonspeech-Gesture effects (V∩G) in both the left (LH) and right (RH) hemispheres (Figure 4A). MNI coordinates for the STS peak conjunction effects were: A∩V LH = −61, −42, 6; A∩V RH = 59, −32, 2; V∩G LH = −49, −52, 10; V∩G RH = 57, −44, 10. Significant conjunction effects for both A∩V and V∩G were observed in the left inferior frontal sulcus and bilateral middle frontal gyrus. Effects specific to A∩V were present in the left temporoparietal junction, while effects specific to V∩G were seen in bilateral visual cortices including hMT, right inferior frontal sulcus, and bilateral precentral sulcus/gyrus. Visual activations in which speech was preferred (V > G) exhibited considerable overlap with A∩V but not with V∩G (Figure 4B), suggesting that multisensory-responsive STS activates preferentially to visual speech. Note that some of the V > G activation on the ventral bank of the STS was due to deactivation in the G condition, rather than large activations in the V condition (see Visual Speech in Figure 3).

#### STS Region of Interest Results

##### GLMM region of interest results

**Figure 5:**
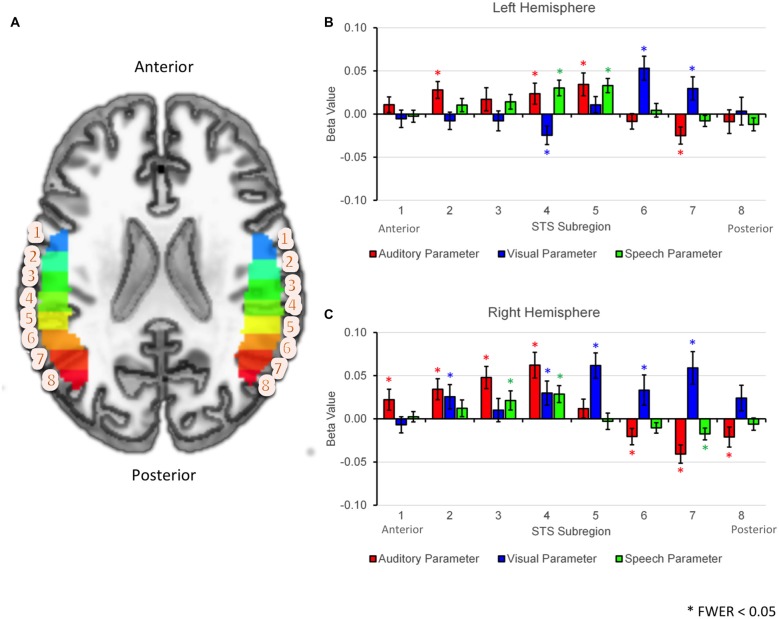
**ROI analyses based on linear mixed effects modeling. (A)** Axial cut-away rendering of the study-specific template in MNI space showing left and right hemispheres overlaid with probabilistically defined STS regions of interest. Individual STS subregions have been color-coded (arbitrary colormap) and numbered 1–8 moving anterior to posterior. **(B,C)** Results of the generalized linear mixed model (GLMM) analysis are plotted by subregion number separately for the left **(B)** and right **(C)** hemispheres. Group average fixed effects of the auditory parameter (AP), visual parameter (VP) and speech parameter (SP) are given by the heights of the red, blue and green bars respectively. Effects are shown separately for each STS subregion (1–8, horizontal axis). Significant effects (FWER corrected *p* < 0.05) are each marked with an asterisk. Error bars reflect 1 SEM.

The results of the GLMM analysis revealed significant positive effects of AP (preference for auditory stimuli) in anterior- or mid-STS subregions (Figure 5; LH: 2, 4, 5; RH: 1–4), while significant positive effects of VP (preference for visual stimuli) were observed primarily in posterior STS subregions (LH: 6–7; RH: 5–7). Overlapping positive effects of AP and VP were observed in two right hemisphere subregions (RH: 2, 4). Significant positive effects of SP (preference for speech vs. nonspeech) were observed in mid-STS subregions in both hemispheres (LH: 4–5, RH: 3–4). The spatial distribution of AP, VP, and SP effects were somewhat different across hemispheres. First, positive effects of AP were smaller or nonsignificant in anterior STS subregions in the left hemisphere, while such effects were larger and consistently significant in the right hemisphere. Second, positive effects of VP extended from posterior to anterior STS subregions in the right but not the left hemisphere. Finally, the transition zone from primarily visual to multimodal activation appeared to localize differently within each hemisphere—subregion 5 in the left hemisphere and subregion 4 in the right hemisphere. However, the broad pattern—namely, a transition from VP to SP/mixed to AP moving posterior to anterior—was maintained across hemispheres.

##### Principal component analysis

**Figure 6:**
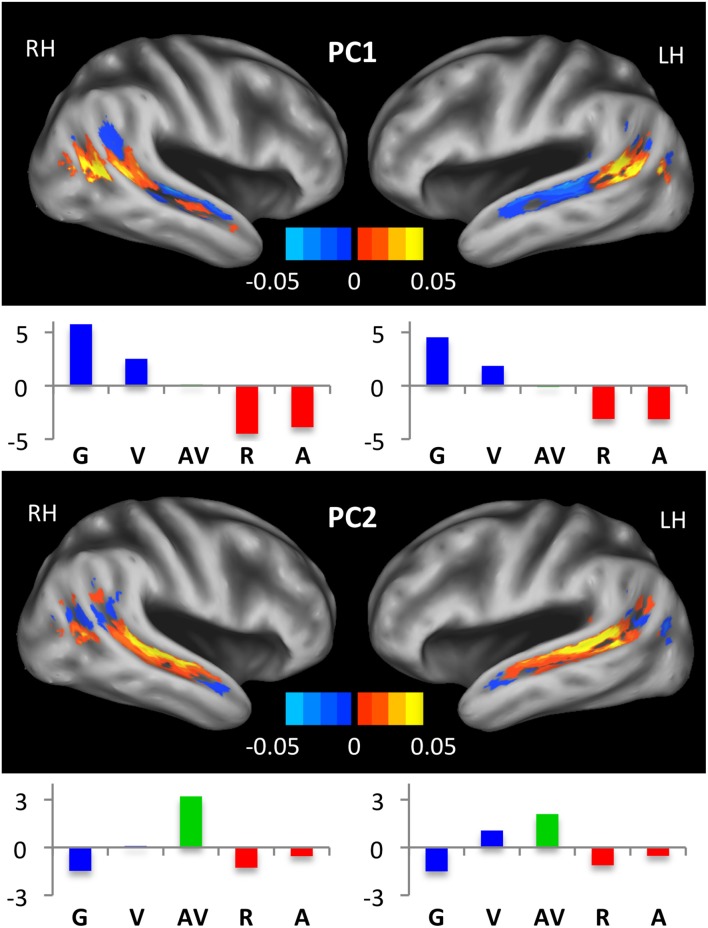
**Principal component maps.** Condition scores and voxel coefficients for the first two principal components are shown. Principal component analyses were performed separately for left and right STS. Voxel coefficients are displayed on inflated surface renderings of the study-specific template. Color maps indicate the sign and magnitude for voxel coefficients. Condition scores are displayed as bar plots beneath the relevant brain image, with conditions color-coded as in Figures 2, 3, 5. Voxels with large positive (negative) coefficients activated preferentially to conditions with positive (negative) scores. For example, positive voxels for the first principal component responded maximally during the G and V conditions, while negative voxels responded most to R and A.

We also used a data-driven approach to capture patterns of activation across the STS. Group mean activations in each of our five experimental conditions and across all voxels of the STS were entered into a principal component analysis considering each voxel as a variable and each condition as an observation. The analysis was performed separately for left and right hemisphere STS ROIs, without splitting into subregions. The first two principal components explained 79.83% and 17.09% of the variance in the left STS, respectively, and 81.96% and 15.80% of the variance in the right STS, respectively. In Figure 6, we list the condition scores and plot the voxel coefficients for each principal component. In both hemispheres, the first principal component (PC1) primarily described activation differences between unimodal auditory (A, R) and unimodal visual (V, G) conditions. As such, large positive condition scores were observed for V and G, while large negative condition scores were observed for A and R. Therefore, voxels that loaded positively on PC1 were “visual-preferring” while voxels that loaded negatively on PC1 were “auditory-preferring.” As can be seen in Figure 6 (top), voxel coefficients transitioned from positive (visual-preferring) in the posterior STS to negative (auditory-preferring) in the anterior STS in both hemispheres, with the positive-negative boundary closely aligned to the posterior-most extent of the Sylvian fissure. This pattern was especially clear in the left hemisphere, whereas visual-preferring voxels in the right hemisphere extended more anteriorly and along the ventral bank of the anterior STS. In both hemispheres, the largest negative coefficients were located on the dorsal bank of the mid-anterior STS, and the largest positive coefficients were located on the ventral bank of the posterior STS.

The second principal component (PC2) was essentially a “multisensory speech” component. In both hemispheres, the condition scores for PC2 were large and positive for AV, followed in order by V, A, R and finally G, which had a large negative condition score. As such, voxels that loaded positively on PC2 preferred multisensory speech, while voxels that loaded negatively on PC2 preferred unisensory (primarily visual) nonspeech. As can be seen in Figure 6 (bottom), large positive voxel coefficients were observed primarily on the dorsal bank of the middle and mid-posterior STS, while negative voxel coefficients were observed mostly in the posterior, visual regions of the STS.

**Figure 7:**
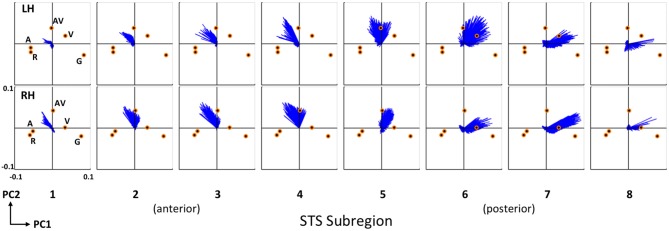
**Condition preferences based on mean activity changes.** Series of PCA biplots spanning all eight STS subregions are displayed for the right (top) and left (bottom) hemispheres. Each PCA biplot shows voxel coefficients as blue vectors, with orange circles representing the scaled principal component scores for each experimental condition. Conditions are labeled on the left-most plots for each hemisphere and these labels apply to the neighboring plots located to the right. On each plot, the first principal dimension is represented along the abscissa and the second principal dimension along the ordinate. The range of the axes (labeled on the bottom left plot) is identical for all 16 plots. Voxel coefficient vectors point toward the condition(s) preferred in terms of mean activity; shorter vectors correspond to voxels that did not exhibit a strong preference. These results clearly demonstrate a functional-anatomic gradient of activation preferences transitioning from visual (subregions 6–8) to audiovisual (subregions 4–5) to auditory (subregions 1–3) moving posterior to anterior.

To further emphasize the transition in voxel activation patterns moving from posterior STS regions to more anterior STS regions, we generated a series of principal component biplots (Figure 7). The biplot is a two-dimensional characterization of voxel activation patterns along the first two principal dimensions (PC1 and PC2). On each biplot, scaled condition scores (orange circles) and voxel coeffcients (blue vectors) are plotted together in the same space. The biplot can be interpreted as follows. Conditions that evoked similar patterns of activation across STS voxels have similar scores, and thus the orange circles corresponding to those conditions will be physically closer to each other on the biplot. A single blue vector represents each voxel and the voxel’s condition preference is given by the direction and magnitude of the vector; that is, the vector will point toward the preferred condition(s) and the length of the vector describes the strength of that preference. We show separate biplots for each STS subregion in the left (Figure 7, top) and right (Figure 7, bottom) hemispheres. In the series of biplots for each hemisphere, we observe a gradual transition from visually-preferring voxels in posterior subregions (6–8) which point toward (i.e., prefer) visual conditions (AV, V, G), to multisensory voxels in mid-STS subregions (4–5) which primarily point toward AV, to auditory-preferring voxels in anterior STS subregions (1–3) which point toward auditory conditions (AV, A, R). Minor differences exist between the hemispheres but the overall pattern is clearly maintained.

##### *Post hoc* analysis of visual STS subregions

In the GLMM analysis (see “GLMM Region of Interest Results” Section) we found that a number of posterior STS subregions were significantly activated by conditions containing visual (facial) motion but not by auditory conditions. In a *post hoc* analysis, we tested whether activation in these posterior subregions differed across our three facial motion conditions (V, AV, G). Although a significant visual effect in the GLMM indicates that (relatively) increased activation was present in all three conditions, differential activation may have emerged for the following reasons: differences in total motion energy in G vs. V, AV (see “Motion Energy in Visual Speech vs. Nonspeech Facial Gestures” Section); effects of bimodal stimulation, i.e., AV vs. V, G; or visual-speech specificity, i.e., V, AV vs. G. In the left hemisphere, significant activation to facial motion had been observed in subregions 6 and 7 (Figure 5, top). A significant difference in activation between the facial motion conditions was observed in subregion 7 (*F*_(2,34)_ = 9.72, *p* < 0.01) but not subregion 6 (*F*_(2,34)_ = 0.33, *p* = 0.63). In the right hemisphere, significant activation to facial motion had been observed in subregions 5–7 (Figure 5, bottom). Significant differences in activation between the facial motion conditions were observed in subregion 7 (*F*_(2,34)_ = 15.27, *p* < 0.001), and subregion 6 (*F*_(2,34)_ = 7.56, *p* < 0.01), but not subregion 5 (*F*_(2,34)_ = 1.01, *p* = 0.36). In both hemispheres, subregions exhibiting significant differences in activation between the facial motion conditions showed a consistent pattern: G > V > AV. This pattern matched the pattern of activation observed in the visual motion area hMT, defined here using a term-based meta-analysis for “visual motion” in NeuroSynth. Thus, the more posterior visual STS subregions were sensitive to motion energy and were (partially) inhibited by multisensory stimulation, mirroring hMT, while the anterior-most visual STS subregions did not respond differentially across the facial motion conditions.

For these anterior-most visual STS subregions (left subregion 6, right subregion 5), we conducted a MVPA analysis using a SVM to determine whether facial motion conditions could be distinguished in terms of the pattern of activation across voxels, whereas no differences were observed in terms of average activation magnitude. We conducted two pairwise classifications: V vs. G, which tested for representational differences between two different classes of facial motion, and V vs. AV, which tested for representational differences between unisensory and multisensory versions of the same facial motion stimulus. In fact, V and G were successfully discriminated on the basis of activation patterns in the left (d′ = 1.26, *t*_(17)_ = 4.16, *p* < 0.01) and right (d′ = 0.99, *t*_(17)_ = 5.00, *p* < 0.01) hemispheres, while V and AV were not successfully discriminated in either hemisphere (both *p* > 0.05). Thus, the anterior-most visual STS subregions did not respond differentially to V and G in terms of overall magnitude, despite differences in total motion energy, but did distinguish these conditions in their patterns of activation across voxels. However, patterns of activation were indistinguishable for V and AV, which contained identical facial motion information.

## Discussion

In the present fMRI study, we set out to answer two questions concerning the organization of multisensory speech streams in the STS: (1) Does activation follow a posterior-to-anterior gradient from facial motion processing regions, to multisensory speech regions, to auditory regions? And if so (2) where along this gradient do speech-specific representations emerge in the STS; in particular, do posterior-visual regions of the STS play a role in speech processing? To answer these questions we presented participants with a variety of speech and nonspeech conditions: auditory speech, visual speech, audiovisual speech, spectrally rotated speech and nonspeech facial gestures. Briefly, we confirmed within a single group of participants that activation in the STS does follow a posterior-to-anterior gradient (visual → multisensory → auditory) as observed across studies in Figure 1. We found that speech-specific representations emerged in multisensory regions of the middle STS (mSTS), but we also found that speech could be distinguished from nonspeech in multivariate patterns of activation within posterior visual regions immediately bordering the multisensory regions.

### A Posterior-to-Anterior Functional Gradient in the STS

Different analysis methods converged to reveal a posterior-to-anterior functional organization of the STS. First, in a whole-brain analysis, we performed the conjunctions V∩G and A∩V, respectively. The logic here was that V∩G should identify voxels that responded to two types (speech and nonspeech) of visual stimuli (i.e., biological/facial motion regions), while A∩V should identify voxels that responded to speech across multiple input modalities (i.e., audiovisual speech regions). Both conjunctions reliably identified voxels in the STS bilaterally. Crucially, we observed that activations to A∩V were located anterior to, and were largely non-overlapping with, activations to V∩G (Figure 4A), providing support for a posterior-anterior functional organization within the STS.

Second, in an anatomical ROI analysis, we used a parameterization approach to examine patterns of activation across eight STS subregions divided evenly along the anterior-posterior axis (see “STS Region of Interest Analysis” Section). An AP coded for increased activation in conditions containing an auditory signal (A, AV and R), while a VP coded for increased activation in conditions containing facial motion (V, AV and G). In accordance with the whole-brain results, we observed significant VP activation in posterior STS subregions bilaterally and significant AP activation in mid- or anterior-STS subregions bilaterally (Figure 5). A clear transition from VP to AP occurred in the mid-posterior STS in both hemispheres.

Third, using a data-driven approach, a principal component analysis revealed that the posterior-anterior distinction between visual and auditory activation explained ~80% of the variance in activation patterns across STS voxels. The PCA was performed on mean activation across participants in each of the five experimental conditions, treating voxels as variables and conditions as observations. The first principal component (PC1), which distinguished maximally between A/R on the one hand (large negative condition scores), and V/G on the other hand (large positive condition scores), loaded positively (i.e., visual activation) on posterior STS voxels and loaded negatively (i.e., auditory activation) on anterior STS voxels. A clear transition from positive- to negative-loading voxels was observed in the mid-posterior STS in both hemispheres (Figure 6, PC1). Visual activations extended slightly more anterior in the right STS, which was also observed in the parameterization analysis. The second principal component (PC2), which distinguished maximally between AV (large positive condition score) and G (large negative condition score), loaded positively (i.e., multisensory-speech activation) on voxels in the middle and mid-posterior STS where visual activation transitioned to auditory activation (Figure 6, PC2). A PCA biplot analysis (Figure 7) demonstrated that activation transitioned gradually from visual, to multisensory, to auditory moving posterior to anterior.

Previous studies have demonstrated that visual speech and nonspeech facial gestures tend to co-activate only the posterior regions of the STS (Campbell et al., 2001; Bernstein et al., 2011), and it has also been demonstrated that visual speech activations diminish moving from posterior to anterior in the STS, while auditory speech activations remain elevated (Wright et al., 2003). Moreover, face-specific functional connectivity has been observed between face-selective regions of the fusiform gyrus and the pSTS (Zhang et al., 2009; Turk-Browne et al., 2010), and both task-based and meta-analytic functional connectivity analyses show coupling between pSTS and V5/MT (Lahnakoski et al., 2012; Erickson et al., 2017). Haxby et al. (2000) suggest that the pSTS is involved in processing changeable or dynamic aspects of faces (Said et al., 2010). Recent evidence suggests that more anterior STS regions, specifically the mSTS, are crucial for auditory speech processing (Specht et al., 2009; Liebenthal et al., 2010; Bernstein and Liebenthal, 2014).

In accordance with these and the present findings, a recent study by Deen et al. (2015) revealed a posterior-to-anterior functional-anatomic organization of the STS using a range of socially relevant stimuli/tasks. Specifically, Deen et al. (2015) found a reliable posterior-to-anterior ordering of task-related response preferences: the posterior-most STS was activated by a theory of mind task, followed by activations to biological motion in the pSTS, activation to dynamic faces in the pSTS, activation to voices in the mSTS, and activation to a language task in the anterior STS. However, unlike in the present study, they found significant overlap between activations within the pSTS that were related to biological-motion, faces, and voices. This may owe to the use of non-speech human vocalizations such as coughing and laughter in their study (see, Stevenson and James, 2009). Alternatively, overlapping face-voice activations may have occurred in what we observe presently as a multisensory “transition zone” in the mid-posterior STS.

The present study has mapped the organization of the STS for multisensory speech processing in more detail than these previous studies. Overall, the results indicate the presence of a posterior-to-anterior functional gradient in the STS moving from facial motion processing, to multisensory processing, to auditory processing.

### Speech-Specific Activations in Middle STS

Having established the existence of a posterior-to-anterior processing gradient in the STS, we wanted to ascertain the locations at which speech-specific activation was present. Before proceeding, we should note that an original goal of this study was to examine speech-specific activation within the auditory and visual modalities separately, namely by using the conditions R and G as within-modality nonspeech controls (see “Group Analysis” Section). However, no voxels in the brain showed greater activation for A than R, and the pattern of activation across STS voxels was extremely similar for A and R (Figure 7). We believe that spectral rotation may have failed to completely remove phonetic information from the speech stimuli (Liebenthal et al., 2005). The result was that “speech-specific” activations were driven primarily by differences between the speech conditions (A, V, AV) and nonspeech facial motion (G).

With that said, our results consistently showed that speech-specific activations emerged in multisensory regions of the middle and mid-posterior STS. In our whole-brain analysis, activation for V > G overlapped strongly with A∩V, but not with V∩G (Figure 4B). In our parameterization analysis, the SP, which coded for increased activation in conditions containing speech (A, V, AV), was significant in bilateral STS subregions in the middle and mid-posterior STS where activation transitioned from visual to auditory along the anterior-posterior axis (Figure 5). Our principal component analysis revealed strong activation preferences for multisensory speech (AV) in this “transition zone” in both hemispheres (Figure 7). Moreover, PC2, which maximally distinguished between multisensory speech (AV) and visual nonspeech (G), loaded most strongly on mSTS voxels in both hemispheres.

A recent fMRI study (Bernstein et al., 2011) employed a rather comprehensive set of visual speech and nonspeech stimuli (but no auditory stimuli), demonstrating that a more anterior region of the left pSTS/pMTG responded preferentially to orofacial visual motion when it was speech-related, while more posterior regions of pSTS responded to orofacial motion whether or not it was speech-related. The authors dubbed the anterior speech-related area the “temporal visual speech area” (TVSA). Bernstein et al. (2011) and Bernstein and Liebenthal (2014) have described the TVSA as a high-level, modal visual area. However, our study shows that visual-speech-specific activations also occur in the multisensory STS. Bernstein and Liebenthal (2014) have suggested that the TVSA feeds directly into speech-related regions of multisensory STS (Stevenson and James, 2009). We are aware of no studies that have established unequivocally the level at which auditory and visual speech signals interact in multisensory STS—specifically, whether multisensory speech signals interact at the phonological level, or if, as others have suggested (Calvert et al., 1999; Skipper et al., 2007; Arnal et al., 2009), the outcome of multisensory integration merely informs phonological mechanisms in other brain regions. Bernstein et al. (2011) and Bernstein and Liebenthal (2014) suggest that speech sounds are categorized downstream in more anterior auditory regions of the STS. In the present study, we did not observe speech-specific activations in anterior subregions of the STS (Figure 5), though it should be noted that our task did not require explicit categorization or discrimination of speech sounds. Moreover, we employed sublexical stimuli whereas other studies indicate that speech-related activations in the anterior STS are most prominent in response to word- or sentence-level stimuli (Scott et al., 2000, 2006; Davis and Johnsrude, 2003; Specht and Reul, 2003; Leff et al., 2008; DeWitt and Rauschecker, 2012). Some of our own work suggests that anterior speech-related activations may reflect higher-level combinatorial processing or extraction of prosody rather than analysis of speech sounds *per se* (Humphries et al., 2001, 2005; Rogalsky and Hickok, 2009; Okada et al., 2010).

### Role of Visual STS Subregions in Speech Perception

We were particularly interested in ascertaining the role, if any, of posterior visual STS subregions in the perception of visual speech. Presently, visual STS subregions are defined as those that showed increased activation to conditions containing facial motion (V, AV, G) relative to auditory-only conditions (A, R), i.e., significant positive effects of VP but not AP in our GLMM analysis (see “GLMM Region of Interest Results” Section). For each of these subregions, we tested for differences in activation across the facial motion conditions. Speech-specific activations (i.e., V, AV vs. G) were not expected given the results of our whole-brain analysis (see “Whole-Brain Results” Section), which demonstrated that visual-speech-specific activation (V > G) was located farther anterior in the STS. Nonetheless, the possibility remained that a more fine-grained analysis of posterior STS subregions would reveal such effects. We were also interested in testing whether visual subregions would be sensitive to differences in total motion energy across conditions (G > [V, AV]). Specifically, we took advantage of the fact that the nonspeech facial gestures in our G stimuli produced more total motion energy than the speech gestures in our V/AV stimuli (see “Motion Energy in Visual Speech vs. Nonspeech Facial Gestures” Section). We wanted to know whether activation in posterior-visual subregions of the STS would increase with total motion energy, as would be expected for canonical visual motion regions, or if activation would be relatively insensitive to low-level motion kinematics. Recent studies suggest that, indeed, activation in the pSTS may be relatively insensitive to motion kinematics, image size, or viewpoint (Lestou et al., 2008; Grossman et al., 2010), and some investigators have suggested that the pSTS codes high-level aspects of biological motion such as action goals or intentions (Pelphrey et al., 2004; Vander Wyk et al., 2009). Therefore, we were most interested in determining which, if any, of the subregions *did not* demonstrate differential activation on the basis of total motion energy (i.e., G > V, AV), and, for these subregions, whether the pattern of activation across voxels would discriminate among the facial motion conditions (V vs. G; V vs. AV).

In fact, we found a significant effect of facial motion condition in several of the posterior visual STS subregions (see “*Post hoc* Analysis of Visual STS Subregions” Section). Namely, activation followed the pattern G > V > AV, which was the same pattern exhibited by a canonical visual motion area, hMT. However, no significant effect of condition was observed for the visual subregion immediately bordering the mSTS “transition zone” in which activation preferences changed from visual to auditory/multisensory (left hemisphere Subregion 6, right hemisphere Subregion 5; Figure 5). In these anterior-most visual pSTS subregions, activation was nearly identical for V, AV and G (*p* > 0.3). Crucially, while these subregions did not distinguish between facial motion conditions in terms of univariate activation, they did distinguish between speech and nonspeech facial motion (V vs. G) in terms of the multivariate pattern of activation across voxels (i.e., using MVPA). Activation patterns were, however, not influenced by presentation modality (V vs. AV), and therefore information coded in the multivariate patterns reflects the class of visual motion stimulus (speech vs. nonspeech). To summarize, these particular visual pSTS subregions: (a) can be distinguished from neighboring visual STS subregions located immediately posterior because they do not show sensitivity to total motion energy; (b) can be distinguished from neighboring multisensory STS subregions located immediately anterior because they do not activate to auditory-only stimuli and do not activate preferentially to visual speech vs. nonspeech gestures; and (c) nonetheless distinguish between visual speech and nonspeech on the basis of multivariate patterns of activation. We therefore conclude that these posterior visual STS subregions immediately bordering the mSTS “transition zone” code for high-level aspects of facial actions, and that speech actions can be distinguished from nonspeech actions on the basis of population-level representations of these high-level features (see also Said et al., 2010).

### Hemispheric Differences

In terms of hemispheric differences, perhaps the most striking pattern observed in the present data is the broad similarity in STS activation preferences across hemispheres. This is most clearly observable in the PCA (Figures 6, 7), which demonstrates very similar patterns of condition scores and per-STS-subregion voxel preferences across hemispheres. However, some subtle differences in hemispheric organization were observed. First, the extent of visual-speech-specific activation (V > G) was greater in the left STS (Figure 5B). This was supported by the results of the GLMM analysis (Figure 5) which demonstrated that the response to VP (which includes nonspeech condition G) was generally larger in mid- and anterior-STS subregions of the right vs. the left hemisphere. The same pattern was revealed in the coefficient maps of PC1 in the PCA (Figure 6, top). Thus, overall, it seems the strength of speech-specific activations in the right hemisphere was lower than in the left hemisphere. This concurs with previous imaging studies investigating effects of intelligibility with visual or audiovisual speech (Callan et al., 2003, 2004; Sekiyama et al., 2003; Okada and Hickok, 2009), and may be generally related to the idea of a “hemispheric lateralization gradient” (Peelle, 2012; Specht, 2013) in which stronger patterns of left-hemisphere lateralization emerge at higher levels of analysis in speech processing (e.g., auditory vs. phonological vs. lexical-semantic or syntactic). The speech-specific activations observed presently could reflect sublexical phonological analysis which, according to lateralization theories, would predict an intermediate level of left hemisphere lateralization. Second, the location of the multisensory “transition zone” was slightly different across hemispheres (left subregion 6/5, right subregion 5/4). We believe this merely reflects differential alignment of our anatomically-defined STS ROIs across hemispheres; the functional pattern is nearly identical. Third, the GLMM analysis revealed stronger auditory activation (AP) in anterior STS subregions of the right vs. the left hemisphere (Figure 5). While we can only speculate as to the reason for this, one possibility is that anterior regions of the right STS perform a more general acoustical (perhaps prosodic) analysis of speech-like signals, while anterior regions of the left hemisphere perform higher-level linguistic analyses. This notion is in line with theories of anterior STS function discussed above (see “Speech-Specific Activations in Middle STS” Section) and with lateralization theories discussed here.

### Conclusion

In the present fMRI experiment, we measured activation to a range of auditory and visual speech (A, V, AV) and nonspeech (R, G) stimuli, focusing particularly on the pattern of activation in the STS. The results demonstrated the following: (1) activation in the STS follows a posterior-to-anterior functional gradient from facial motion processing, to multisensory processing, to auditory processing; (2) speech-specific activations arise in multisensory regions of the middle STS; (3) abstract representations of visible facial gestures emerge in visual regions of the pSTS that immediately border the multisensory regions. We therefore suggest a functional-anatomic workflow for speech processing in the STS—namely, lower-level aspects of facial motion are processed in the posterior-most visual STS subregions; high-level/abstract aspects of facial motion are extracted in the pSTS immediately bordering mSTS; visual and auditory speech representations are integrated in mSTS; and integrated percepts feed into speech processing streams (Hickok and Poeppel, 2007; Rauschecker and Scott, 2009), potentially including auditory-phonological systems for speech sound categorization in more-anterior regions of the STS (Specht et al., 2009; Liebenthal et al., 2010; Bernstein and Liebenthal, 2014).

## Author Contributions

JHV and GH conceptualized and designed the research. JHV, DM and KS prepared the stimuli. JHV and DM collected the data. JHV, KIV and FR analyzed the data. JHV wrote the manuscript. All authors interpreted the data, provided critical feedback and revised the manuscript.

## Funding

During this investigation, JHV was supported by the National Institute on Deafness and Other Communication Disorders (National Institutes of Health, NIH) Award DC010775 from the University of California, Irvine, CA, USA. The investigation was supported by the National Institute on Deafness and Other Communication Disorders Award DC03681 to GH.

## Conflict of Interest Statement

The authors declare that the research was conducted in the absence of any commercial or financial relationships that could be construed as a potential conflict of interest.

## Footnotes

^1^We will use the terms “audiovisual”, “multisensory” and “multimodal” to describe brain regions that respond to more than one sensory modality. We will not test directly for multisensory *interactions* using criteria established in the animal electrophysiological literature (see, Stevenson et al., 2014; Venezia et al., 2015). Therefore, our use of the terminology will not strictly distinguish between regions that respond to two different unimodal signals vs. regions that *prefer* multimodal stimulation.

^2^http://www.vislab.ucl.ac.uk/cogent_2000.php

^3^http://afni.nimh.nih.gov/afni
